# Cyclopædia of the Practice of Medicine, Vol V

**Published:** 1876-02

**Authors:** 


### Books Reviewed.

*Cyclopedia of the Practice of Medicine.* Edited by Dr. H. Von Ziemssen. Vol. V. Diseases of the Respiratory Organs. By Profs. Juergensen, Hertz, Ruhle and Rindfleish. A. H. Buck, M. D., American Editor. New York: W. W. Wood & Co. 1875.

The subjects treated in this volume are Croupous Pneumonia, Catarrhal Pneumonia, Hypostatic Processes in the Lungs, Pneumonia from Embolism, by Prof. Juergensen. Anæmia, Hyperæmia and Œdema of the Lungs, Hemorrhages, Atelectasis, Atrophy, Hypertrophy, Pulmonary Emphysema, Gangrene of the Lungs, New Growths in the Lungs and Mediastinum, and Parasites of the Lungs, by Prof. Hertz. Pulmonary Consumption and Acute Miliary Tuberculosis, by Prof. Ruhle. Chronic and Acute Tuberculosis, by Prof. Rindfleish.

In Croupous Pneumonia, Juergensen shows that the greatest danger is from the failure of the heart to perform its functions. One of the most active agents in producing this failure of the heart's action is the high temperature accompanying the disease. The author therefore advises the employment of means which shall reduce the temperature. Cold bathing is freely employed, and quinia administered in large doses, as high as fifteen grains being given to a child under one year, at one dose, and seventy grains to an adult.

Prof. Ruhle enumerates among the causative factors of tuberculosis, scrofula, and anything which depresses the general health, either hereditary or acquired. Hæmorrhage is not considercd as productive of tuberculosis, but the author holds that when the blood is retained and decomposes, it may hasten a morbid process already commenced. The author also attaches considerable importance to the form of the chest in the cause of consumption, or perhaps we should say as one of the diagnostic signs by which its presence or probable danger may be suspected. It certainly seems that this is an important point for examination, especially where consumption has been present in other members of the family.

Rindfleisch teaches that scrofula and tuberculosis are identical; that the tubercular poison is located in the scrofulous glands; that when these glands become inflamed, local foci for the generation of tubercle is formed. The volume is full of interest and is fully up to the standard of those already published.

